# Prevalence of major depressive disorder and its associated factors among adult patients with neurolathyrism in Dawunt District, Ethiopia; 2022: community-based cross-sectional study

**DOI:** 10.1186/s12888-024-05755-7

**Published:** 2024-04-16

**Authors:** Melaku Bimerew, Teshome Gebremeskel, Biruk Beletew, Wondye Ayaliew, Mulugeta Wodaje, Manay Ayalneh

**Affiliations:** 1 Department of Pediatric and Child Health Nursing, College of Medicine and Health Science, Injibara University, Injibara, Ethiopia; 2 Department of Human Anatomy, College of Health Science, Woldia University, Woldia, Ethiopia; 3 Department of Nursing, College of Health Science, Woldia University, Woldia, Ethiopia; 4 Department of Plant Biotechnology, Woldia University, Woldia, Ethiopia; 5 Department of Midwifery, College of Health Science, Woldia University, Woldia, Ethiopia

**Keywords:** MDD, Neurolathyrism, Prevalence, Ethiopia

## Abstract

**Introduction:**

Major Depressive Disorder (MDD) is one of the commonest mental disorders affecting more than 250 million people globally. Patients with chronic illnesses had higher risks for developing MDD than the general population. Neurolathyrism is a chronic illness characterized by lifelong incurable spastic paralysis of lower extremities; causing permanent disability. It is highly prevalent in Dawunt district, Ethiopia; with a point prevalence of 2.4%. Despite this, there were no previous studies assessing the prevalence of MDD among patients with neurolathyrism in Ethiopia.

**Objective:**

To assess the prevalence of MDD and to identify its associated factors among patients with neurolathyrism in Dawunt district, Ethiopia.

**Methods:**

A community based cross-sectional study was conducted on 260 samples in Dawunt district from February 01 to March 30/ 2021. Multistage sampling technique was used to select study participants. The patient Health Questionnaire-9 (PHQ-9) depression screening tool was used to diagnose MDD. PHQ-9 is a standardized depression screening tool and a PHQ-9 score of ≥ 10 has a sensitivity and specificity of 88.0% [95% CI (83.0–92.0%)] and 85.0% [95% CI (82.0–88.0%)] for screening MDD. Data were collected by interview; entered to EpiData version 4.2.0; exported to SPSS version 25.0 for analysis; descriptive statistics and binary logistic regression model were used; AOR with 95% CI was used to interpret the associations; and finally results were presented by texts, charts, graphs, and tables.

**Results:**

A total of 256 adult patients with neurolathyrism were participated; and the prevalence of MDD was found to be 38.7%. Being female [AOR = 3.00; 95% CI (1.15, 7.84)], living alone [AOR = 2.77; 95% CI (1.02–7.53)], being on neurolathyrism stage-3 [AOR = 3.22; 95% CI (1.09, 9.54)] or stage-4 [AOR = 4.00; 95% CI (1.28, 12.48)], stigma [AOR = 2.69; 95% CI (1.34, 5.39)], and lack of social/ family support [AOR = 3.61; 95% CI (1.80, 7.24)] were found to have statistically significant association with an increased odds of MDD; while regular exercise and ever formal counselling were found to have statistically significant association with a decreased odds of MDD.

**Conclusion:**

The prevalence of MDD among neurolathyrism patients in Dawunt district was high. Lack of social support, stigma, not getting formal counselling, and not involving in regular exercise were modifiable risk factors. Therefore, social support, reducing stigma, formal counselling, and encouraging regular exercise might help to reduce the burden of MDD among neurolathyrism patients.

## Introduction

Depression is an episodic or recurrent mental disorder [1, 2] mainly characterized by the following nine self-reported symptoms: depressed mood or hopelessness, little interest or pleasure on doing things, poor appetite or overeating, insomnia or hypersomnia, feeling of tired or lack of energy, low self-esteem or feeling of excessive guilt or worthlessness, inattention or lack of concentration, slowness or hyperactivity, and thoughts that would be better off dead or suicidal attempt. It is also characterized by an impairment of social, occupational, or other areas of functioning without any apparent causes. According to the fifth edition of the diagnostic and statistical Manual for Mental Disorders (DSM-5), an individual having at least five out of the above nine self-reported symptoms with in the last 2 weeks; with one of the five symptoms being “depressed mood, hopelessness, or feeling down” OR “little interest or pleasure on doing things”; can be diagnosed as having major depressive disorder [3–5].

Major depressive disorder (MDD) is among the most common psychiatric disorders affecting more than 250 million people worldwide [6]. In western communities, an estimated lifetime prevalence of MDD ranges from 12 to 16% [7]. Furthermore, despite the advancement of medical technologies, infrastructures, and living conditions, the prevalence of MDD is increasing over the past decades. In the United States, prevalence of MDD was increased from 8.1 to 15.8% between 2009 and 2019 [8]. In Africa, more than 29 million people suffer from depression and MDD is common among patients with chronic illness [9]. Studies from Nigeria revealed that the prevalence of MDD among patients with chronic illness to be 32.5% [10]. The prevalence of MDD was also reported to be as high as 34.8% among patients with chronic illness and 63.1% in traumatic patients [11]. In sub-Saharan African countries, pooled results reporting the lifetime prevalence of MDD are limited [12]. Single studies from Uganda [13], Sudan [14], and Tanzania [15] had revealed the prevalence MDD to be 29.3%, 31%, and 42.4% among youths, traumatic adults, and patients with chronic illness respectively.

Even if MDD is a very treatable disorder, it is a serious health problem. People with this disorder did not recognize that they are sick; and they will not get help or seek treatment. It increases the risk of suicidal behaviour [16]; and it is the main contributing factor for preventable immature mortality worldwide. It also increases health care cost [17]. In 2018, it was estimated that the health care cost patients with MDD reaches about $326 billion in the United States [18]. It also reduces the economic growth of a country by reducing working ability and productivity. Moreover, it reduces immunity and increases the risk of infection [19]. Generally, MDD is a leading cause of disability and poor quality of life [20].

On the other hand, individuals with physical disabilities have an increased risk of developing MDD than the general population; with an estimated average prevalence reaching 29–41% [21]. Neurolathyrism is a neurological disease of the upper motor neuron characterized by a chronic irreversible spastic paralysis (physical disability) of lower extremities resulting from over consumption of grass pea. Patients with this disease have no treatment; and will have lifelong disabilities; and might be more prone to MDD [22, 23]. Authors of this article believe that neurolathyrism and MDD have complex interactions. Neurolathyrism causes lifelong and incurable paralysis, decreased working ability, self-care deficit, and dependency; which in turn might increase the prevalence of MDD. MDD on the other had is a major cause of disability worldwide [20]. Therefore, patients with neurolathyrism might suffer from a double burden of disabilities both from neurolathyrism and depression.

In Dawunt district, Ethiopia, neurolathyrism is highly prevalent; affecting about 2.4% of the population [24]. Despite this, there are no previous studies conducted to assess the prevalence of depression and/ or MDD among patients with neurolathyrism in the district; and also in the country. Assessing the prevalence of MDD among neurolathyrism patients and identifying its associated factors is vital to increase quality of life of neurolathyrism patients through provision factor oriented interventions. Therefore, this study was aimed to assess the prevalence of MDD and its associated factors among patients with neurolathyrism in Dawunt district, Ethiopia; and will help to develop factor oriented strategies for reducing the prevalence of MDD among neurolathyrism patients in the country.

## Methods

### Study area, design, and period

This study was community-based cross-sectional study conducted in Dawunt district, North Wollo Zone, Ethiopia in 2022. The district had an estimated population of 80,211; living in 18,653 households. It has also 15 administrative Kebeles; of which about 11 kebeles are grass pea cultivation areas. In those grass pea cultivation areas, neurolathyrism is highly prevalent with an estimated population prevalence of 2.4% (95% CI = 2.0–3.0%) [24]. Therefore, this study was part of a previous study conducted to assess the prevalence and psychosocial impacts of neurolathyrism in the district that was conducted from February 01 to March 30/ 2021 [24]; and this part was aimed to assess the prevalence of MDD and its associated factors among adult patients with neurolathyrism in this district.

### Population and eligibility criteria

All adults (individuals aged 18 years and above) living in Dawunt district and who has been diagnosed as having neurolathyrism were the source population; and all adults having neurolathyrism and living in selected Kebeles of the district were the study population for this study. But, individuals who were critically ill, have hearing problems and other communication problems, and live in the district for less than 6months were excluded from the study.

### Sample size and sampling technique

Sample size was determined by using single population proportion formula by considering the following assumptions: prevalence of depression in the general population in Ethiopia = 11.0%, confidence interval = 95%, margin of error = 5%, design effect = 1.5 and non-response rate = 15%; giving a total sample size of 260. Samples were selected by using multistage sampling technique. First, by considering the rule of thumb, 5 Kebeles out of the total 11 grass pea cultivation Kebeles were selected by simple random sampling technique. Then, households having neurolathyrism patients were identified by house-to-house screening; and all households having neurolathyrism patients were included. Then, for households having more than one neurolathyrism patient, simple random sampling was used to select one participant.

### Outcome variable and measurement

The outcome variable of this study was Major Depressive Disorder (MDD). The patient Health Questionnaire-9 (PHQ-9) depression screening tool was used to identify individuals having MDD. PHQ-9 is a standardized depression screening tool and a PHQ-9 score of ≥ 10 has a sensitivity and specificity of 88.0% [95% CI (83.0–92.0%)] and 85.0% [95% CI (82.0–88.0%)] for screening MDD. It has nine questions. Each question would be rated from 0 to 3; in which 0 being “not at all”, 1 being “for several days”, 2 being “for more than half the days”, and 3 “for being nearly every day”. According to PHQ-9, MDD can be initially diagnosed if an individual have at least five of the following nine problems with in the last 2weeks; of which one of the five problems being “Little interest or pleasure in doing things for more than half the days” OR “Feeling down, depressed, or hopeless for more than half the days”: (1) Little interest or pleasure in doing things for more than half the days; (2) Feeling down, depressed, or hopeless for more than half the days; (3) Trouble falling or staying asleep, or sleeping too much for more than half the days; (4) Feeling tired or having little energy for more than half the days; 4) Poor appetite or overeating for more than half the days; (5) Feeling tired or having little energy for more than half the days; (6) Feeling bad about self for more than half the days; (7) Trouble concentrating on things for more than half the days; (8) Moving or speaking so slowly or being so fidgety or restless for more than half the days; (9) thoughts that would be better off dead, or of hurting self for several days. Additionally, presence of functional impairment and exclusion of other causes for those nine problems are required to diagnose MDD [25]. Therefore, in this study, MDD was diagnosed by considering the above listed criteria. On the other hand, individuals having two up to four of the above nine problems were considered as having other depressive disorder.

### Independent variables and measurement

Perceived stigma was measured by using the stigma scale for chronic illness-short form (SSCI-8). This scale has 8 questions; each question is rated from 1 to 5; giving a total score of 8–40. Individuals having a score of > 8 were considered as having some form of stigma [26].

Social or family support was measured by using the Oslo social support scale [27]. For the ease of analysis, individuals having moderate to strong social support (score of 9–14) were grouped as “have social support”; and individuals with poor social support (score of 3–8) were grouped as “have no/ lack social support”.

Stage of neurolathyrism was determined clinically by considering individuals having mild disability with no need of sticks as stage-1; individuals having moderate disability requiring support with one stick as stage-2; individuals having severe disability requiring support with two sticks as stage-3; and individuals having very severe disability with inability to move with supporting sticks (crawler stage) as stage-4 [28].

Regular exercise was assessed by interviewing; and an individual involving in a planned physical exercise for at least 75 min per week was considered as he/she was involving in regular exercise [29].

Substance abuse was measured by using the CAGE-AID questionnaire which is scored from 0 to 4. An individual having a score > 0 was considered as a substance abuser [30].

### Data collection method, tool, and procedure

Data was collected by face to face interview using interviewer administered questionnaire adapted from previous articles and the patient health questionnaire-9 (PHQ-9) depression screening tool [25]. First, 2 physicians were involved to identify neurolathyrism patients by clinical diagnosis using the following criteria: having symmetrical spastic paralysis of lower extremities with intact sensory perception AND walking on the balls of the feet with lurching and scissoring type gait AND sign/ symptom begins after and during consumption of grass pea AND clinical exclusion of other causes of paralysis [24, 28]. Then the interview of adult neurolathyrism patients was conducted by 4 BSc Nurses. The whole process of data collection was supervised by 2 MPH professionals.

### Data quality control, data analysis, and presentation

Pre-test of the questionnaire, training of the data collectors, close supervision, coding, double data entry, and cross-tabulation were used to control the data quality. After cleaning and coding, data were entered to EpiData version 4.2.0 and exported to SPSS version 25 for analysis. Descriptive statistics were employed to describe the sociodemographic and other related variables of the study participants. Binary Logistic Regression model was used to identify factors associated with Major depressive disorder. First, bivariable logistic regression was employed to identify the possible explanatory variables; and variables with p-value of less than 0.25 were considered as candidates for multivariable logistic regression. Then, multivariable logistic regression was employed to identify statistically significant variables. Hosmer and Lemeshow goodness of the fit was used to test model fitness; and correlation matrix was used to test multicolinearity between independent variables. Adjusted odds ratio (AOR) with 95% confidence interval was used to interpret the association; and p-value of less than 0.05 was used to declare statistically significant association. Finally, results were presented using texts, tables, and graphs.

## Results

### Sociodemographic characteristics of the study participants

A total of 256 adults having neurolathyrism were participated in this study with a response rate of 98.46%. Most of the participants (87.50%) were males (Table 1). The participants’ age was ranged from 18 to 65years; and the mean age was 35.48 ± 13.71years. The neurolathyrism onset age was ranged from 4 to 37years with a mean onset age of 17.32 ± 7.91years. Among participants, the minimum duration with neurolathyrism was 4years and the maximum was 40years; with a mean duration of 17.95 ± 8.28years.

**Table 1:** Sociodemographic characteristics; and bivariable and multivariable logistic regression showing the factors associated with major depressive disorder among adults with neurolathyrism in Dawunt district, Ethiopia: 2022

Variables			Frequency	Percentage	MDD		COR (95%CI)	AOR (95%CI)
					Yes	No		
**Sex**		Female	32	12.50	17	15	1.96 (0.93–4.14)*	3.00 (1.15–7.84)**
		Male	224	87.50	82	142	1	
**Current age**		18-24years	65	25.39	28	37	1.17 (0.61–2.26)	
		25-34years	74	28.91	23	51	0.70 (0.36–1.35)	
		35-44years	33	12.89	15	18	1.29 (0.57–2.90)	
		45^+^years	84	32.81	33	51	1	
**Neurolathyrism onset age**		< 10years	39	15.23	18	21	1.22 (0.48–3.10)	
		10-19years	129	50.39	42	87	0.69 (0.32–1.50)	
		20-29years	54	21.09	25	29	1.23 (0.52–2.93)	
		30^+^years	34	13.28	14	20	1	
**Duration with Neurolathyrism**		30^+^years	34	13.28	16	18	1.93 (0.74–5.03)*	1.43 (0.31–6.67)
		20-29years	64	25.00	25	39	1.39 (0.59–3.25)	1.35 (0.40–4.56)
		10-19years	120	46.88	46	74	1.35 (0.62–2.93)	1.50 (0.55–4.10)
		< 10years	38	14.84	12	26	1	
**Marital status**		Divorced	49	19.14	30	19	2.89 (1.45–5.75)*	2.10 (0.89–5.00)
		Widowed	11	4.30	2	9	0.41 (0.08–1.97)	0.39 (0.07–2.29)
		Single	80	31.25	26	54	0.88 (0.48–1.61)	1.12 (0.48–2.60)
		Married	116	45.31	41	75	1	
**Educational status**		Secondary & above	24	9.38	12	12	1.74 (0.74–4.13)*	1.61 (0.53–4.88)
		Primary	73	28.52	29	44	1.15 (0.65–2.03)	1.32 (0.61–2.86)
		Less than primary	159	62.11	58	101	1	
**Occupation**		No occupation	41	16.02	14	27	0.82 (0.37–1.82)	0.47 (0.16–1.38)
		Farmer	140	54.69	56	84	1.06 (0.60–1.88)*	0.46 (0.18–1.21)
		Non-farmer	75	29.30	29	46	1	
**Residence**		Rural	223	87.11	90	133	1.81 (0.80–4.06)*	2.61 (0.80–8.53)
		Urban	33	12.89	9	24	1	
**Living alone**		Yes	32	12.50	20	12	3.06 (1.42–6.58)*	2.77 (1.02–7.53)**
		No	225	87.89	80	145	1	
**Stage of Neurolathyrism**		Stage 4	32	12.50	18	14	3.38 (1.48–7.73)*	4.00 (1.28–12.48)**
		Stage 3	35	13.67	18	17	2.78 (1.25–6.18)*	3.22 (1.09–9.54)**
		Stage 2	91	35.55	36	55	1.72 (0.94–3.17)*	1.99 (0.90–4.38)
		Stage 1	98	38.28	27	71	1	
**Perceived stigmatized**		Yes	162	63.28	76	86	2.73 (1.55–4.79)*	2.69 (1.34–5.39)***
		No	91	35.55	23	71	1	
**Lack Social or Family Support**		Yes	75	29.30	41	34	2.56 (1.47–4.44)*	3.61 (1.80–7.24)****
		No	181	70.70	58	123	1	
**Lifelong/ chronic comorbidities**		Yes	50	19.53	26	24	1.97 (1.06–3.68)*	1.74 (0.78–3.89)
		No	206	80.47	73	133	1	
**Substance Abuse**		Yes	32	12.50	17	15	1.96 (0.93–4.14)*	2.19 (0.77–6.23)
		No	224	87.50	82	142	1	
**Involve in regular exercise**		No	226	88.28	92	134	2.26 (0.93–5.48)*	3.20 (1.07–9.62)**
		Yes	30	11.72	7	23	1	
**Had ever get formal counselling about Neurolathyrism**		No	196	76.56	84	112	2.25 (1.18 (4.31)*	2.38 (1.06–5.34)**
		Yes	60	23.44	15	45	1	
**Know that neurolathyrism is not curable**		Yes	218	85.16	88	130	1.66 (0.78–3.52)*	1.80 (0.68–4.77)
		No	38	14.84	11	27	1	
**Income category**		Low	182	71.09	73	109	1.28 (0.50–2.81)	
		Medium	42	16.41	15	27	1.06 (0.40–2.78)	
		High	32	12.50	11	21	1	
**Main income Source**		Farming	230	89.84	90	140	1.61 (0.31–8.46)	
		Marketing	19	7.42	7	12	1.46 (0.22–9.62)	
		Employment	7	2.73	2	5	1	
**Family history of depressive symptoms**		Yes	11	4.30	6	5	1.96 (0.58–6.61)	
		No	245	95.70	93	152	1	
**Have a family member affected by Neurolathyrism**		Yes	19	7.42	11	8	2.33 (0.90–6.01)*	2.48 (0.69–8.92)
		No	237	92.58	88	149	1	

AOR: Adjusted Odds Ratio; COR: Crude Odds Ratio; MDD: Major Depressive Disorder

*Significant at p-value < 0.25 in the bivariable logistic regression

**Significant at p-value < 0.05 in the multivariable logistic regression

***Significant at p-value < 0.01 in the multivariable logistic regression

**** Significant at p-value < 0.001 in the multivariable logistic regression

Regarding to their marital status, about 45.31% of the participants were married and about 19.14% were divorced. More than half (54.69%) of the participants were farmers and for the majority of the participants (89.84%) farming was their main income source. About 87.11% of the participants were rural residents and about 12.50% were living alone. About 38.28%, 35.55%, 13.67%, and 12.50% of the participants were on stage-1, stage-2, stage-3, and stage-4 of neurolathyrism respectively. About 14.84% of the respondents did not know that neurolathyrism is incurable. About 7.42% and 4.30% of the respondents had a family member affected by neurolathyrism and a family history of depressive symptoms respectively (Table 1).

### Prevalence of major depressive disorder and other depressive disorders

**Fig. 1:**
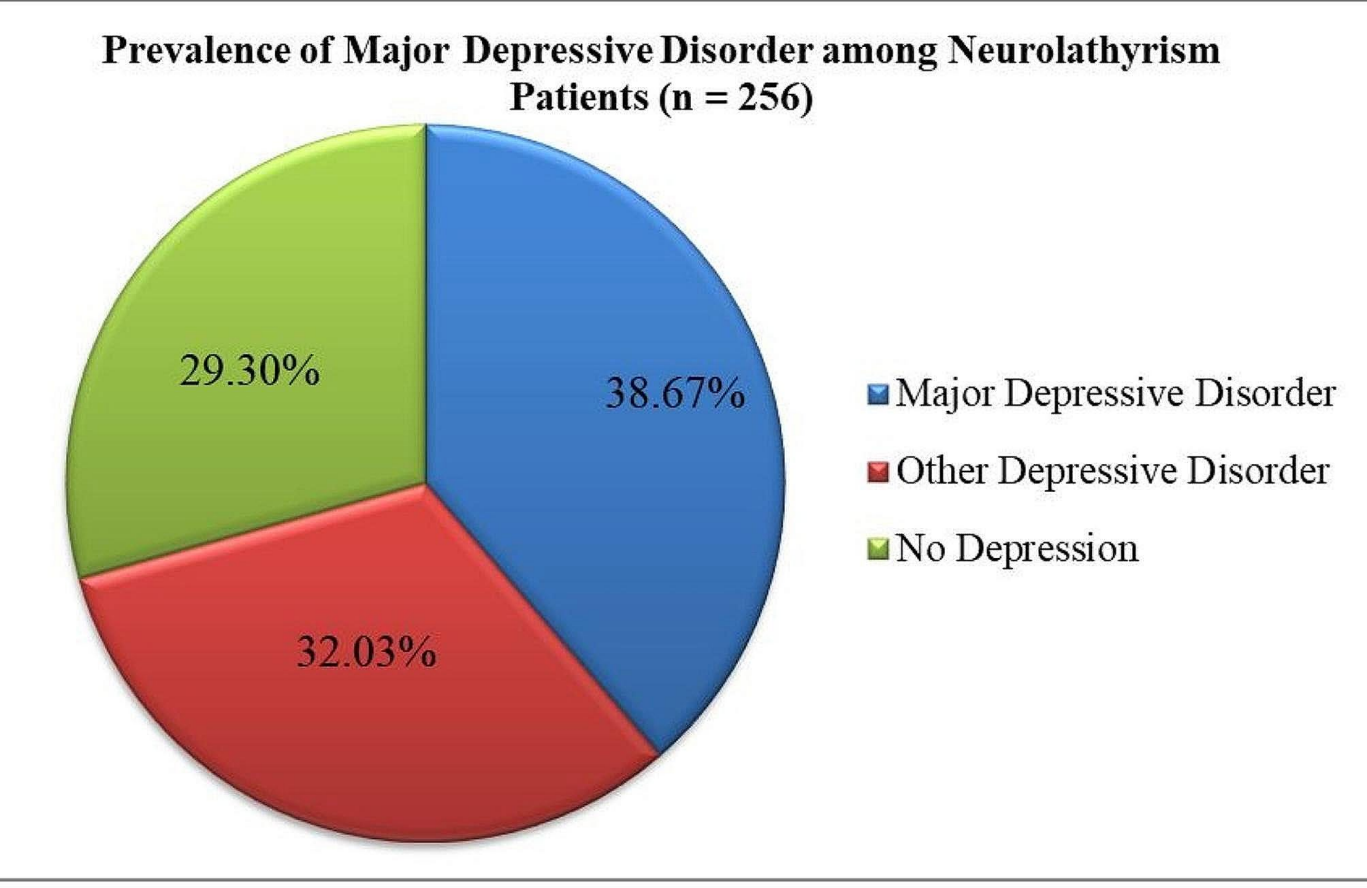
PHQ-9 based Prevalence of Major Depressive Disorder among Adults with Neurolathyrism in Dawunt, Ethiopia: 2022

**Fig. 2:**
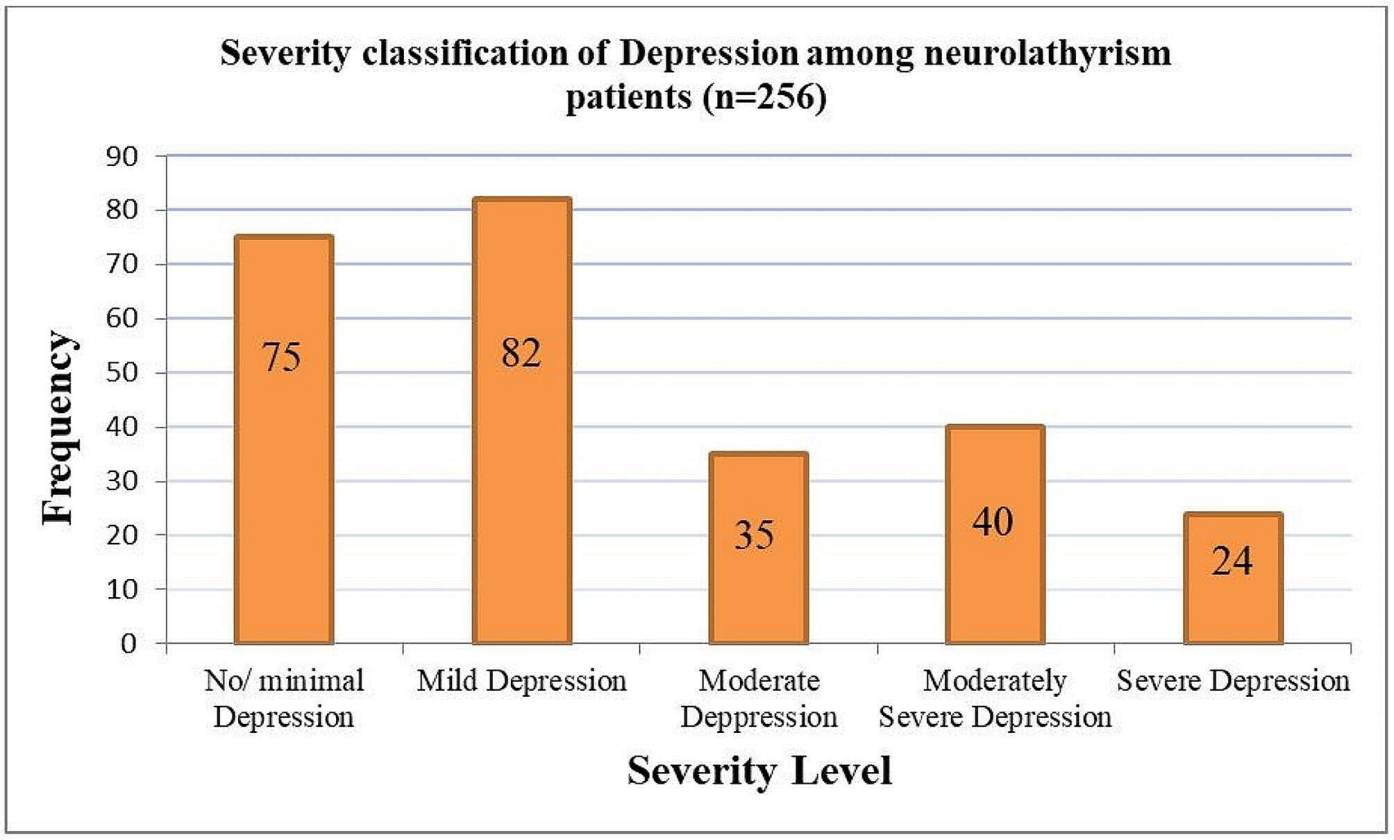
PHQ-9 based Severity Classification of Depression among Adults with Neurolathyrism in Dawunt district, Ethiopia: 2022

According to the PHQ-9, about 181 (70.70%) of adults with neurolathyrism had depression; in which 99 (38.67%) had major depressive disorder (MDD); and 82 (32.03%) had other depressive disorder (Fig. 1). Regarding to the severity of depression, about 24 (13.26%; *n* = 181) of the depressive patients had severe depression; and 40 (22.10%) had moderately severe depression (Fig. 2). Almost all (98.90%) of patients who have depressive symptoms did not seek treatment for their symptoms.

### Factors associated with MDD among Neurolathyrism patients

A total of 21 independent variables were entered to the bivariable logistic regression model; and variables with a p-value of < 0.25 were considered to be candidates of the multivariable logistic regression model. In the bivariable logistic regression model, five variables (participants’ current age, neurolathyrism onset age, income category, main income source, and family history of depressive symptoms) were associated with the outcome variable with a p-value of ≥ 0.25; and were not candidates for the multivariable logistic regression model. As a result, 16 variables (sex, marital status, educational status, residence, living alone, duration with neurolathyrism, neurolathyrism stage, perceived stigma, lack of social support, presence of lifelong or chronic comorbidities, substance abuse, involving in regular exercise, ever getting formal counselling on neurolathyrism, knowing that neurolathyrism is incurable, family history of depressive symptoms, having a family member affected by neurolathyrism) were entered to the multivariable logistic regression; and 7 variables (sex, living alone, neurolathyrism stage, perceived stigma, lack of social support, involving in regular exercise, and ever getting formal counselling on neurolathyrism) were found to have statistically significant association (p-value < 0.05) with the outcome variable (Table 1).

Females with neurolathyrism were 3.00 times more likely to have major depressive disorders than males with neurolathyrism [AOR = 3.00; 95% CI (1.15, 7.84); p-value = 0.025)]. Neurolathyrism patients who live alone were 2.77 times more likely to have major depressive disorder than individuals who lives with families or relatives [AOR = 2.77; 95% CI (1.02, 7.53); p-value = 0.046)]. Individuals who were on stage-4 and stage-3 neurolathyrism were 4.00 times [AOR = 4.00; 95% CI (1.28–12.48); p-value = 0.017)] and 3.22 times [AOR = 3.22; 95% CI (1.09, 9.54); p-value = 0.035)] more likely to have major depressive disorder respectively than individuals with stage-1 neurolathyrism (Table 1).

Neurolathyrism patients who did not involve in regular exercise were 3.20 times more likely to have major depressive disorder than who did involve in regular exercise [AOR = 3.20; 95% CI (1.07, 9.62); p-value = 0.038)]. The odds of major depressive disorder was 2.69 times higher in neurolathyrism patients who perceived as they were stigmatized than who did not had perceived stigma [AOR = 2.69; 95% CI (1.34, 5.39); p-value = 0.005)]. Neurolathyrism patients who did not ever get formal counselling about neurolathyrism were 2.38 times more likely to have major depressive disorder than who did ever get formal counselling [AOR = 2.38; 95% CI (1.06, 5.34; p-value = 0.036)]. Lastly, neurolathyrism patients who lack social or family support were 3.61 times more likely to have major depressive disorder than individuals having moderate to strong social or family support [AOR = 3.61; 95% CI (1.80, 7.24); p-value < 0.001)] (Table 1).

## Discussion

This study was aimed to assess the prevalence of major depressive disorder (MDD) and to identify its associated factors among patients with neurolathyrism in Dawunt district, Ethiopia using the PHQ-9 depression screening tool. A community based cross-sectional study design was employed; a total of 256 adults with neurolathyrism were participated; and the prevalence of MDD in those patients was found to be 38.7%. The result of this study was higher than a prior community based study conducted in Ebinat town, Ethiopia; as that study had reported the prevalence of depression among Ethiopian adults to be 17.5% [31]. This discrepancy might be due to differences in the study population; as that study was used the general adults, while this study used adults with chronic illness or neurolathyrism. It was also higher than a study conducted in Nigeria among patients with chronic illness (diabetes mellitus); as that study had revealed the prevalence of MDD in diabetic adults to be 32.5% [10]. This difference might be due to differences in depression screening tool (that study was used Hamilton rating scale for depression (HAM-D) and/ or differences in the type of the chronic illness. But the result of this study was nearly similar with findings from Tanzania (42.4%) [15] and South Africa (34.8%) [32]. On the other hand, the result of this study was much lower than another study from South Africa (63.1%) [11]which might be due to differences in the study participants (that study involves only females) and/ or the depression screening tool (that study uses the BDI depression screening tool).

In this study, Females with neurolathyrism were found 3 times more likely to be affected by MDD than males. Prior studies from Ethiopia had also reported that females were more at risk than males for depression [31, 33]. Studies from United States [8], Taiwan [34], and Nigeria [35] had also revealed similar findings. In contrary to this, studies from Ethiopia [36] and Burkina Faso [37] had reported as there was no association between sex and MDD. In fact females have fluctuating hormonal levels which affect their mood and leads to depression [38]. With this biological difference, if females are exposed to another risk factor for depression like chronic illnesses, they would have greater risk of developing MDD than males. Another possible explanation for increased odds of depression among females than males might be their difference in expressing feelings. Males tend to hide their feeling; remains to seem strong; and might not express their depressive symptoms. On the other hand, females can easily express their feelings; seek help; and tend to tell their depressive symptoms for others including for researchers [39]. Genetic differences might also have a role; even if there are no sufficient evidences explaining this issue [40].

Patients who were on stage-4 neurolathyrism were 3.4 times more likely to have MDD than patients on stage-1 neurolathyrism. Similarly, the odds of MDD among neurolathyrism patients who were on stage-3 were 2.8 times higher than patients who were on stage-1 neurolathyrism. Since there were no previous studies assessing the prevalence of MDD among neurolathyrism patients, comparing results regarding to the association between stages of neurolathyrism and MDD was difficult. A study conducted in Ethiopia [41] had reported that severity level of disability was not associated with depression; which was incongruent with the result of this study; while a study from Ohio, Cleveland [42] had showed as there was an association between disease severity and MDD. But authors of this article believe that severity (stage) of neurolathyrism ranges from mild disability to the crawler stage. Individuals who are on latter stages (stage-3 and stage-4) would have higher extent of functional disability, higher rates of self-care deficit, and complete dependency than the earlier stages of neurolathyrism; which might expose them to MDD; and this might explain the exhibited association between MDD and stages of neurolathyrism.

Neurolathyrism patients who perceived as they had been stigmatized were 2.7 times more likely to develop MDD than patients who did not perceived as they had been stigmatized by the community. Studies from China [43], Saudi Arabia [44], and South India [45] had also reported that stigma can increase the odds of depression. Similarly, neurolathyrism patients who did not have social or family support were also 3.6 times more likely to have MDD than patients who had moderate to strong social or family support. Prior studies from Ethiopia had also reported similar findings. Studies from Ghana [46] and United Kingdom [47] had also revealed that poor social support could increase the prevalence of depression. In fact, illnesses that cause functional or physical disability like neurolathyrism predispose patients to self-care deficit. With this self-care deficit, lack of a supporter to meet self-care needs could result in depression. Furthermore, neurolathyrism patients who live alone were almost 2.8 times more likely to have MDD than neurolathyrism patients who live with their families or relatives. Preceding studies from Ethiopia [31] and India [48] had reported similar findings. The association between living alone and depression has been reported by numerous articles. Indeed, living alone leads to decreased contact with others or social isolation. This isolation might in turn predispose to depressive symptoms; and this might explain the unveiled association between living alone and MDD in this study.

Individuals who did not involve in regular exercise were 3.2 times more likely to develop MDD than individuals who did involve in regular exercise for at least 75 min per week. This implies that doing a planned exercise for at least 75 min per week can decrease MDD almost by 69% among patients with neurolathyrism. This result was in line with previous literature as they had reported that regular exercise could alleviate symptoms of depression; and it might have antidepressant effect. Randomized control trials, systematic reviews, and meta-analyses had recommended regular moderate to high intensity aerobic exercise as a single, adjuvant, or a combination therapy for reducing and preventing depressive symptoms. Regular exercise could increase feeling of worth fullness and self-esteem. Moreover, it has an anti-inflammatory and anti-oxidant effect; reducing neuroinflammation and oxidative stress that are assumed to have an effect on the pathogenesis of depression [49–52]. Despite this anti-depressant effect of exercise, only few of the participants of this study were involved in doing regular exercise.

Patients who did not ever get Formal counselling about neurolathyrism were 2.4 times more likely to have MDD than patients who had ever got formal counselling. This implies that if health care workers provide counselling about neurolathyrism, the prevalence of MDD among neurolathyrism patients might be reduced by 58%. Studies assessing the association between MDD and formal counselling on neurolathyrism among neurolathyrism patients are null. Hence, comparison of this result with other findings was difficult. But Authors of this article believe that counselling on neurolathyrism for patients with neurolathyrism would help them to acquire adaptation and self-adjustment that might reduce the odds of MDD.

### Strength and limitations of the study

Even if neurolathyrism is common in grass pea cultivation areas of the country, there were no previous studies assessing the prevalence of MDD and its associated factors among neurolathyrism patients in Ethiopia. Therefore, being the first study is one of the strengths. Robust research methods were employed. But, this research might not be free from limitations. First, as there were no previous studies conducted on this title, comparison of findings were difficult; and this might make the discussion somewhat shallow. Second, as this was a cross-sectional study, it could not show cause-effect relationship between the risk factors and MDD. Lastly, some of the associated factors had somewhat wider confidence interval; which might be due to smaller observed values.

### Conclusion and recommendations

The prevalence of major depressive disorder among adults with neurolathyrism in Dawunt district was found to be high. Being female, living alone, being on neurolathyrism stage-3 or stage-4, stigma, and lack of social/ family support were found to have statistically significant association with an increased odds of MDD; while involving in regular exercise and ever getting formal counselling about neurolathyrism were found to have statistically significant association with a decreased odds of MDD. Therefore, supporting neurolathyrism patients or creating a social support system, family or social reintegration, community education towards reducing stigmatization of neurolathyrism patients, encouraging and providing education about the importance of regular exercise for neurolathyrism patients, and providing formal counselling about neurolathyrism for patients with great emphasis for females and neurolathyrism stage-3 and stage-4 patients might be helpful to reduce the prevalence of MDD among neurolathyrism patients. Further studies with advanced research methods, wider study areas, and larger sample sizes might also be crucial.

## Data Availability

No datasets were generated or analysed during the current study.
